# RISK FACTORS FOR POST-LIVER TRANSPLANT BILIARY COMPLICATIONS IN THE ABSENCE OF ARTERIAL COMPLICATIONS

**DOI:** 10.1590/0102-672020200003e1541

**Published:** 2020-12-18

**Authors:** Agnaldo Soares LIMA, Bárbara Buitrago PEREIRA, Sven JUNGMANN, Carla Jorge MACHADO, Maria Isabel Toulson Davison CORREIA

**Affiliations:** 1 Alfa Institute of Gastroenterology, Hospital das Clínicas, Federal University of Minas Gerais (UFMG), Belo Horizonte, MG, Brazil; 2 Department of Surgery, Faculty of Medicine, UFMG, Belo Horizonte, MG, Brazil; 3 Residence in Clinical Medicine, Hospital das Clínicas, UFMG, Belo Horizonte, MG, Brazil; 4 Founderslane, Health Berlin, Berlin, Germany; 5 Department of Preventive and Social Medicine, Faculty of Medicine, UFMG, Belo Horizonte, MG, Brazi

**Keywords:** Liver transplantation, Bile Ducts, Cytomegalovirus infections, Transplante de fígado, Ductos biliares, Infecções por citomegalovirus

## Abstract

**Background:**

- Biliary complications (BC) represent the most frequent complication after liver transplantation, up to 34% of cases.

**Aim:**

To identify modifiable risk factors to biliary complications after liver transplantation, essential to decrease morbidity.

**Method:**

Clinical data, anatomical characteristics of recipient and donors, and transplant operation features of 306 transplants with full arterial patency were collected to identify risk factors associated with BC.

**Results:**

BC occurred in 22.9% after 126 days (median) post-transplantation. In univariate analyses group 1 (without BC, n=236) and group 2 patients (with BC, n=70) did not differ on their general characteristics. BC were related to recipient age under 40y (p=0.029), CMV infection (p=0.021), biliary disease as transplant indication (p=0.018), lower pre-transplant INR (p=0.009), and bile duct diameter <3 mm (p=0.033). CMV infections occurred sooner in patients with postoperative biliary complications vs. control (p=0.07). In a multivariate analysis, only CMV infection, lower INR, and shorter bile duct diameter correlated with BC. Positive CMV antigenemia correlated with biliary complications, even when titers lied below the treatment threshold.

**Conclusions:**

Biliary complications after liver transplantation correlated with low recipient INR before operation, bile duct diameter <3 mm, and positive antigenemia for CMV or disease manifestation. As the only modifiable risk factor, routine preemptive CMV inhibition is suggested to diminish biliary morbidity after liver transplant.

## RESUMO

**Racional:**

Complicações biliares (CB) são os eventos adversos mais frequentes após o transplante de fígado, ocorrendo em até 34% dos procedimentos.

**Objetivo:**

Identificar fatores de risco modificáveis para o aparecimento de complicações biliares após transplantes de fígado, essenciais para diminuir morbidade.

**Método:**

Investigação dos dados clínicos, características anatômicas de receptores e doadores e informações sobre a operação de 306 transplantes com artéria hepática pérvia, para identificar fatores de risco associados ao aparecimento de CB.

**Resultados:**

CB ocorreu em 22,9% após 126 dias (mediana) do transplante. Em análise univariada pacientes do grupo 1 (sem CB, n=236) e grupo 2 (com CB, n=70) não diferiram em suas características gerais. CB esteve relacionada à idade do receptor menor que 40 anos (p=0,029), infecção pelo citomegalovírus (CMV, p=0,021), doença biliar como indicação ao transplante (p=0,018), RNI pré-transplante mais baixo (p=0,009) e diâmetro do ducto biliar <3 mm (p=0,033). Infecções pelo CMV ocorreram mais precocemente em pacientes com CB (p=0,07). Na análise multivariada, somente infecção por ele, INR mais baixo e menor diâmetro do ducto biliar mantiveram correlação com CB. Antigenemia positiva para CMV correlacionou com CB mesmo em títulos inferiores ao cutoff para tratamento.

**Conclusões:**

CB após transplante hepático esteve relacionada com menores RNI do receptor antes da operação, diâmetro do ducto biliar <3 mm e antigenemia ou manifestação clínica positiva para CMV. Como único fator de risco evitável, tratamento preemptivo para inibição do CMV é sugerido para diminuir morbidade biliar após o transplante.

## INTRODUCTION

Biliary complications are frequent in patients who undergo deceased donor orthotopic liver transplantation. They occur mainly during the first year after transplantation, more often in the first three months, with an incidence between 11% and 34%^2^ ^,^ ^22^. The frequency of these complications has important implications for the patients’ morbidity and further pressures overburdened health services.

The most common biliary complications include stenosis, occlusion and fistula. They are primarily associated with hepatic artery lesions, such as stenosis and thrombosis^11^. Therefore, biliary complications are not restricted to patients with arterial alterations, which suggests the presence of other risk factors for the development of such complications. Recent research investigated the relationships of further potential risk factors. The most commonly cited of those include: 1) type of anastomosis^15^ ^,^ ^23^; 2) warm and cold ischemia time^5^ ^,^ ^30^; 3) type of preservation solution^20^ ^,^ ^30^; 4) transplantations with ABO incompatibility^17^ ^,^ ^24^; 5) age of the donor and recipient^8^ ^,^ ^19^ ^,^ ^25^ ^,^ ^29^; 6) receptor’s model for end-stage liver disease (MELD) score^30^; 7) hepatitis C recurrence^13^; and 8) cytomegalovirus (CMV) infection^7^ ^,^ ^12^ ^,^ ^14^. 

The aim of this research was to identify modifiable risk factors to biliary complications after liver transplantation, essential to decrease morbidity. 

## METHOD

This retrospective study used data from the medical records of patients undergoing liver transplantations between January 2007 and December 2015 at the Hospital of the Federal University of Minas Gerais (Hospital das Clínicas da Universidade Federal de Minas Gerais), Belo Horizonte, MG, Brazil. The data collection for the analysis was authorized by the Research Ethics Committee of the Federal University of Minas Gerais (CAAE: 64319717.2.0000.5149). During the study period, 496 transplants were performed at the institution, but only cases with patients older than 18 years, transplanted with deceased donor whole liver and followed up for at least six months after the procedure were considered (n=327 cases; 65.9% of total). The data were extracted from the Zeus^®^ electronic health record, in which all findings and interventions before and after the transplantation were recorded.

The standard surgical technique for biliary reconstruction in transplantation is end-to-end biliary anastomosis, with a continuous suture using polydioxanone (PDS) 6.0 or 7.0. Surgeons were free to choose variations in the technique to accommodate the anatomy of the graft and recipient. Patients with primary biliary tract disease (primary or secondary sclerosing cholangitis) and cases of significant disproportion of diameter between the bile duct of the donor and recipient were reconstructed by Roux-en-Y hepaticojejunostomy. Biliary anastomoses were performed after arterial graft reperfusion.

Biliary complications were defined as being identified through diagnostic tests (magnetic resonance cholangiography and ERCP) and whose presence required an endoscopic, percutaneous or surgical intervention. The transplanted patients were divided into four groups: 1) absence of biliary and arterial complications (n=236); 2) biliary complications without arterial complications (n=70); 3) arterial complications without biliary complications (n=8); and 4) biliary and arterial complications (n=13). In these two latter cases, retransplantation was common, rendering a long-term observation of many of these cases impossible. Thus, only groups 1 and 2 (n=306) were analyzed in this study. Biliary complications occurred in 22.9% of patients.

Group 1 and 2 were compared by demographic and clinical aspects of the recipient and donor, as well as by the technical aspects of the operation. Potential risk factors were the age range of the recipient and donor, gender at birth of the recipient and donor, occurrence of CMV infection, transplant indication, MELD score used for selection, severity of liver disease using the MELD score calculated, blood group, laboratory tests of the donor (AST, ALT, sodium, HCO_3_and base excess - BE), cause of brain death of the donor, preservation solution used, diameter of the bile duct of the graft (≤3 mm or >3 mm), cold ischemia time (CIT), and biliary artery ischemia time (BAIT). CIT was the time interval between vascular clamping in the donor to portal reperfusion in the recipient, and BAIT was the time interval between portal reperfusion and arterial reperfusion in the recipient.

The cases were divided into patients and donors aged below 40 years or 40 years and older for comparison by age group. CMV infection was considered to be present when antigenemia (pp65) results or the presence of symptoms suggestive of cytomegalic infection led to ganciclovir treatment. Asymptomatic patients who displayed antigenemia with weak positivity (i.e. ≤2 leukocytes/100,000) were monitored with weekly serial exams and did not receive treatment.

### Statistical analyses

The categorical variables are presented according to frequency and were compared using the chi-square test. For numerical variables we evaluated the distribution of normality (Kolmogorov-Smirnov test), expressed as the mean and standard deviation for normally distributed data or median and interquartile range (IQR) if data were not normally distributed. Comparisons of non-normal distribution variables were performed using the Mann-Whitney and Kruskal-Wallis tests. Variables with a normal distribution were compared using Student’s t-test. In all cases, differences were considered relevant for a level of significance lower than 5% (p<0.05). All comparisons that produced significant differences were included in a multivariate analysis with binary logistic regression, in addition to variables of clinical importance, whose comparison showed a difference with a significance level below 20% (p<0.2). These variables formed an initial multivariate model in which those whose level of significance did not reach a level lower than 5% (p<0.05) by the Wald test were deleted sequentially. The results with a trend towards statistical significance (p<0.10) are reported. For the regression analysis, odds ratios, 95% confidence intervals and p-values are reported. The data were analyzed using Stata for Mac, version 12.

## RESULTS

### Univariate analysis

The sample comprised 219 men (71.6%) and 87 women (28.4%), with a median age of 53.6 years (IQR 44.4-60.2). The indication for transplantation was hepatocyte cirrhosis (viral, alcoholic, or cryptogenic) in 239 cases (78.1%), biliary in 30 cases (9.8%), autoimmune cirrhosis in 19 cases (6.2%) and other etiologies in 18 cases (5.9%). In 57 cases (18.7%), patients with various chronic liver diseases had hepatocellular carcinoma as the main indication for transplantation. The grafts were obtained from male donors in 62.7% of cases, with a median age of 35.6 (IQR 22.9-47.2), the majority being victims of trauma (45.8%) or stroke (41.0%).

The median follow-up time was 2221 days (IQR 1528.5 - 2903.5), and biliary complications occurred in a median of 126 days (IQR 41.8 - 300.8), equivalent to medians of approximately six years and four months, respectively. Among patients in group 2 (n=70), 67 developed stenosis of the bile duct, and 10 patients developed a biliary fistula, seven of which were associated with stenosis and three isolated. The median time to diagnosis of stenosis was 131 days (IQR 48.0 - 329.0), and the median time to diagnosis of fistula was 73.5 days (IQR 18.8 - 149.3). Other clinical and demographic characteristics of the sample are provided in Table 1.

**TABLE 1:** Variables of the recipient, donor and intraoperative period

	All cases n=306 *	Group 1 n=236 (77.1%)	Group 2 n=70 (22.9%)	p
Recipient variables				
Age range				0.029
<40 years	56 (18.3%)	37 (15.7%)	19 (27.1%)	
≥40 years	250 (81.7%)	199 (84.3%)	51 (72.9%)	
Sex [M/F]	219 (71.6%)/ 87 (28.4%)	168 (71.2%)/ 68 (28.8%)	51 (72.9%)/19 (27.1%)	0.786
CMV IgG (reagent/non-reagent)	258 (92.1%)/ 22 (7.9%)	204 (92.7%)/16 (7.3%)	54 (90.0%)/6 (10.0%)	0.587
CMV infection	51 (16.7%)	33 (14.0%)	18 (25.7%)	0.021
Diagnostic group				0.018
Autoimmune	19 (6.2%)	17 (7.2%)	2 (2.9%)	
Biliary	30 (9.8%)	18 (7.6%)	12 (17.1%)	
Hepatocytes	239 (78.1%)	190 (80.5%)	49 (70.0%)	
Other	18 (5.9%)	11 (4.7%)	7 (10.0%)	
Indication group				0.042
Autoimmune	19 (6.2%)	17 (7.2%)	2 (2.9%)	
Biliary	30 (9.8%)	18 (7.6%)	12 (17.1%)	
HCC	57 (18.6%)	44 (18.6%)	13 (18.6%)	
Hepatocytes	182 (59.5%)	146 (61.9%)	36 (51.4%)	
Other	18 (5.9%)	11 (4.7%)	7 (10.0%)	
Allocation MELD	20.0 (17.0-24.0)	20.0 (17.0-24.0)	20.0 (17.0-23.0)	0.412
Calculated MELD	18.0 (15.0-22.0)	18.0 (15.0-23.0)	17.5 (15.0-21.0)	0.459
Bilirubin (mg/dl)	2.9 (1.8-5.0)	2.8 (1.8-5.0)	3.3 (1.8-6.0)	0.482
INR	1.7 (1.4-2.1)	1.7 (1.5-2.1)	1.6 (1.4-1.9)	0.009
INR (categorical)				
< 1.5	89 (29.1%)	61 (25.9%)	28 (29.1%)	0.029
1.5 a 2.5	168 (54.9%)	139 (58.9%)	29 (41.4%)	
> 2.5	49 (16.0%)	36 (15.6%)	13 (18.6%)	
Creatinine (mg/dl)	0.9 (0.7-1.2)	0.9 (0.7-1.2)	0.9 (0.7-1.2)	0.997
Blood group				
A	129 (42.2%)	96 (40.7%)	33 (47.1%)	0.404
AB	12 (3.9%)	9 (3.8%)	3 (4.3%)	
B	28 (9.2%)	25 (10.6%)	3 (4.3%)	
O	137 (44.8%)	106 (44.9%)	31 (44.3%)	
Rh factor				
Rh negative	43 (14.1%)	31 (13.1%)	12 (17.1%)	0.397
Rh positive	263 (85.9%)	205 (86.9%)	58 (82.9%)	
Donor variables				
Donor age range				0.145
<40 years	178 (58.2%)	132 (55.9%)	46 (65.7%)	
≥40 years	128 (41.8%)	104 (44.1%)	24 (34.3%)	
Gender [M/F]	192 (62.7%)/ 114 (37.3%)	145 (61.4%)/ 91 (38.6%)	47 (67.1%)/ 23 (32.9%)	0.386
AST (U/L)	57.0 (35.0-101.5)	58 (35.0-101.0)	50.5 (37.0-109.0)	0.598
ALT (U/L)	40.0 (29.0-71.0)	40.0 (28.0-71.0)	48.0 (31.0-65.0)	0.173
HCO3 (mEq/l)	21.6 ± 4.4	21.7 ± 4.3	20.9 ± 4.6	0.221
Base excess (mmol/l)	-3.5 ± 4.8	-3.3 ± 4.8	-4.2 ± 4.7	0.204
Sodium (mEq/l)	147.3 ± 10.7	147.3 ± 11.0	147.4 ± 9.6	0.968
Surgical Procedure Variables				
Cold ischemia time (min)	501.5 (431.3-640.0)	499.5 (429.9-631.0)	509.6 (434.7-663.0)	0.510
Biliary artery ischemia time (min)	46.0 (39.3-60.8)	47.0 (40.0-61.0)	46.0 (35.0-60.0)	0.445
Preservation solution				
Celsior	51 (17.4%)	42 (18.4%)	9 (13.8%)	0.740
HTK	95 (32.4%)	72 (31.6%)	23 (35.4%)	
IGL1	34 (11.6%)	25 (11.0%)	9 (13.8%)	
UW	113 (38.6%)	89 (39.0%)	24 (36.9%)	
Bile duct diameter				0.033
≤3 mm	33 (13.0%)	21 (10.6)	12 (21.4)	
>3 mm	221 (87.0%)	177 (89.4)	44 (78.6)	
Type of anastomosis				0.772
end-to-end biliary	256 (93.4%)	200 (93.0%)	56 (94.9%)	
hepaticojejunostomy	18 (6.6%)	15 (7.0%)	(5.1%)	

* Not all data are available for some variables

End-to-end biliary anastomoses were performed in 93.4% of cases and Roux-en-Y hepaticojejunostomy was performed in 6.6% of cases. The type of surgical technique used for anastomosis did not indicate a difference in the incidence of biliary complications (p=0.772).

CMV infection occurred within 35 days after surgery (IQR 20.0 - 53.5). Considering each study group separately, the group without biliary complications (group 1) showed infection by CMV in the median period of 39 days (IQR 25 - 56), while the group with biliary complications (group 2) showed a median of 22 days (IQR 16 - 37, Table 1). The comparison of the two groups showed a trend towards an earlier occurrence of infection in patients with biliary complications (p=0.07, Table 2).

**TABLE 2:** Time interval between transplantation and cytomegalovirus infection

	All cases n=306	Group 1 n=236 (77.1%)	Group 2 n=70 (22.9%)	p
Tx-CMV interval (days)	35.0 (20.0 - 53.5)	39.0 (25.0 - 56.0)	22 (16.0 - 37.0)	0.07

Patients who showed positive CMV antigenemia (n=51) were divided into two subgroups according to the need for treatment with ganciclovir. There was no difference between the treated and untreated groups regarding the incidence of biliary complications (p=0.226). However, when the group of patients without CMV blood manifestation was compared with the group of patients with positive antigenemia at low titers (no indication for treatment with ganciclovir), there was a higher incidence of biliary complications in the latter group (p=0.040). The treatment of CMV infection with intravenous ganciclovir was associated with a lower incidence of biliary complications, for values that can be considered similar to those of patients without infection, even with a trend towards statistical significance (p=0.091, Table 3).

**TABLE 3:** Distribution of patients with and without biliary complications according to CMV antigenemia and treatment with ganciclovir

		CMV antigenemia			
		Positive		Negative	
		Ganciclovir (A)	Without treatment (B)	Without treatment (C)	Total
Biliary complication	Present (%)	14 (31.8%)	4 (57.1%)	52 (20.4%)	70
	Absent (%)	30 (68.2%)	3 (42.9%)	203 (79.6%)	236
	Total	44	7	255	306

A vs. B - p = 0.226. B vs. C - p = 0.040 - Odds Ratio 5.205 Confidence Interval 95% 1.13 - 23.98. A vs. C - p = 0.091

### Multivariate analysis

For the multivariate analysis, the following variables were included: transplant indication group (p=0.042), diagnostic group (p=0.018), age range of the donor (p=0.145), age range of the recipient (p=0.029), CMV infection (p=0.021), recipient INR (p=0.009), and bile duct diameter ≤3 mm (p=0.033). The variables CMV infection, receptor INR, and bile duct diameter remained as factors associated with the occurrence of biliary complications (Table 4).

**TABLE 4:** Multivariate analysis including variables presenting with significant differences when comparing patients with or without biliary complications

Variables	OR (CI 95%)	p
INR (ref: 1.5 a 2.5)		
< 1.5	2.2 (1.1; 4.2)	0.020
> 2.5	1.4 (0.5; 3.7)	0.470
CMV infection (ref: absence)	2.6 (1.3; 5.2)	0.007
Bile duct diameter (ref: <3 mm)	0.44 (0.20; 0.98)	0.046

OR=odds ratio; CI95% = 95% confidence interval *p-value <0.05

A recipient INR <1.5 immediately before transplantation was associated with an increased risk of biliary complications compared to a pre-transplant INR between 1.5 and 2.5 (OR=2.2; p=0.020). No differences were observed for INR >2.5 and INR 1.5-2.5 (OR=1.4; p=0.470).

CMV infection was positively associated with the occurrence of biliary complications (OR=2.6; p=0.007). Finally, a bile duct diameter >3 mm was negatively associated with the biliary complications. The odds of biliary complications when the diameter was greater than 3 mm was 56% lower than those with a diameter ≤3 mm (OR=0.44; p=0.046).

## DISCUSSION

Complications that occur after surgical procedures are often attributed to technical issues. However, the techniques of biliary anastomoses are insufficient to explain the frequent cases of stenosis and fistulas, which are the most common complications of liver transplants, affecting up to 34% of patients^2^. It is possible that there are different factors involved in the genesis of these events after liver transplantation; however, the results found in the literature are varied and contradictory. Building on the large number of potential factors that have been associated with biliary complications and the variety of results obtained, we offer a large cohort of patients which received organs of young donors to contribute to clarify this challenging subject^6^ ^,^ ^9^.

Variations of the surgical technique have been tested in an attempt to reduce the incidence of biliary stenosis and fistulas^18^. For some authors, choledochojejunostomy was related to a greater frequency of complications (especially anastomotic stenosis) compared to end-to-end biliary anastomosis^15^ ^,^ ^23^. In this study, this difference was not observed.

The clinical status and demographic characteristics of donors are frequently suspected causes in the development of biliary complications. Feng et al.^8^, showed that donor age >60 years was related to a higher number of biliary complications and lower survival of the graft. Other authors confirmed the relationship of older donor age with the incidence of biliary complications^19^ ^,^ ^25^ ^,^ ^27^ ^,^ ^29^, some of which differentiated anastomotic from non-anastomotic stenosis. The mean age of our younger donors (median=35.6 years) seems to be different than that European (only 58% are below 50 years) and American donors^1^ ^,^ ^27^. However, in this study, neither the donor nor recipient age remained a factor associated with biliary complications after multivariate analysis.

In this study, the etiology of liver disease did not influence the appearance of alterations in biliary drainage. Regarding laboratory tests, a lower INR value of the recipient, immediately before transplantation, was identified as a risk factor (p=0.020) for subsequent development of biliary complications. This finding was not present in any other study. However, the retrospective nature of the present investigation does not allow us to elucidate the causes for this relationship.

CMV infection and its relationship with a higher occurrence of biliary complications have been the subject of discussion^3^ ^,^ ^7^ ^,^ ^9^ ^,^ ^10^. In this sense, the high percentage of individuals serologically positive for CMV in Brazil stands out - a prevalence of 80 to 100% - compared to a prevalence of 40 to 60% in developed countries^21^ ^,^ ^26^ ^,^ ^31^. However, the nature of this relationship is controversial, possibly due to the different forms used to detect infection - some studies use clinical criteria while others use antigenemia positivity or viral material detection via blood sample or liver biopsy (Polymerase Chain Reaction)^16^. Standardization of CMV detection in the biliary tract could yield more consistent results^3^. Gotthardt et al. ^10^ identified CMV infection as a risk factor using the identification of viral DNA in the bile of transplanted patients as a detection method. In this study, multivariate analysis indicated a higher frequency of CMV infection in individuals who developed biliary complications (p=0.007). In addition, subgroups of patients with positive antigenemia were compared, and patients with positive antigenemia, treated or not, had the same incidence of biliary complications. Therefore, the innovation we introduce to the present knowledge concerns patients with positive antigenemia but below the cut-off point to indicate treatment. This subset had more biliary complications when compared to patients with negative antigenemia. This observation suggests value in preemptive treatment with ganciclovir in all patients or, at least, for any positive antigenemia level found after transplantation, to prevent such complications. Biliary complications have not been researched in publications involving prophylaxis and preemptive treatment^12^. Specifically, no comparison was found with a subgroup of patients with pp65 antigenemia or PCR-positive low titers and its relation to biliary complications. 

Limitations of this study include its retrospective nature, which could lead to information bias and can show correlation but not provide evidence of causation. However, posttransplant complications were prospectively recorded in a specific transplantation software system, which increases the reliability of the data obtained. Another limitation was the use of pp65 antigenemia as the tool to detect CMV instead of viremia-detecting methods.

The identification of these factors may help hepatic transplantation teams take preemptive steps to reduce the impact of early biliary complications. Pretransplant INR and bile duct graft diameter are noncontrollable factors, however, their presence should prompt closer monitoring for biliary complications to trigger early interventions. CMV manifestations, on the other hand, are modifiable. This study justifies the use of ganciclovir for all patients with positive antigenemia for CMV, including cases that are below the cut-off values that usually warrant preemptive treatment. Alternatively, early use of everolimus, a known CMV inhibiting immunosuppressive agent should be considered^4^ ^,^ ^28^. However, further investigation may be required to confirm that the presence of the cytomegaly virus is a risk factor for biliary complications.

## CONCLUSION

In our population, three important risk factors were associated with biliary complication: 1) low recipient INR immediately before transplantation; 2) bile duct diameter ≤3 mm; and 3) the occurrence of any title of positive antigenemia for CMV or disease manifestation in the first six months after transplantation. 
